# 基因治疗开启血友病B患者“临床治愈”序章

**DOI:** 10.3760/cma.j.cn121090-20250304-00111

**Published:** 2025-05

**Authors:** 磊 张

**Affiliations:** 中国医学科学院血液病医院（中国医学科学院血液学研究所），国家血液系统疾病临床医学研究中心，天津 300020 National Clinical Research Center for Blood Diseases, Institute of Hematology & Blood Diseases Hospital, Chinese Academy of Medical Sciences & Peking Union Medical College, Tianjin 300020, China

## Abstract

血友病B是一种单基因遗传病，主要表现为全身各部位的自发性或外伤后出血。当前国内对于血友病B的治疗策略主要是因子替代治疗，患者终身面临频繁静脉穿刺。近年来，血友病B基因疗法发展迅速。在血友病B基因疗法即将进入中国临床之际，中华医学会血液学分会血栓与止血学组和中国血友病协作组联合制定了《中国血友病B腺相关病毒载体基因治疗临床应用指导意见（2025）》，为血友病B基因疗法全流程的规范化给出了详细的指导意见。本文将针对《指导意见》形成的背景、核心要点和临床价值做介绍，借以提高国内对基因治疗的认知和重视度。

## Abstract (en)

Hemophilia B is a monogenic inherited disorder characterized by spontaneous or trauma-induced bleeding. In China, the primary treatment strategy for hemophilia B is factor Ⅸ (FⅨ) replacement therapy, requiring patients to undergo frequent venipunctures throughout their lifetime. In recent years, significant advancements have been made in gene therapy for hemophilia B. As gene therapy for hemophilia B is nearing clinical implementation in China, the Thrombosis and Hemostasis Group of the Chinese Society of Hematology of the Chinese Medical Association and the Hemophilia Treatment Center Collaborative Network of China have jointly developed the “Chinese guidance for clinical application of Adeno-associated virus vector-based gene therapy for hemophilia B (2025)”, offering comprehensive recommendations for standardizing the entire gene therapy process. This article will introduce the background, key points, and clinical value of the guidelines to enhance awareness and emphasis on gene therapy for hemophilia B in China.

血友病B是一种编码凝血因子Ⅸ（FⅨ）的基因突变导致血液中FⅨ缺乏而引起的单基因遗传病，主要表现为全身各部位的自发性或外伤后出血[1]。医学界对于血友病B治疗的探索经历了漫长的过程[2]–[3]。上世纪五、六十年代最早期的主要治疗手段是输注血浆，但其FⅨ浓度较低而无法满足临床治疗需求。上世纪六十年代以后，凝血酶原复合物（PCC）进入临床以及之后血源性FⅨ的使用大大提高了血友病B的治疗效果，但也带来了血源传播性病毒感染的风险。上世纪八、九十年代，随着基因克隆技术的进步，重组FⅨ又一次将血友病B的替代治疗带到新的高度，至今已衍生出标准半衰期和延长半衰期的多种FⅨ药物。此外，基于再平衡原理的非因子替代治疗的问世再次推动了血友病B因子替代治疗不足的改善。

然而，因子替代疗法的主要不足在于FⅨ水平存在波动、需要终身接受静脉注射、可能产生抑制物及引发过敏风险，部分患者因依从性差仍出现关节受累加重或自发性出血。非因子替代治疗也需要终身治疗。重型血友病B患者的生活质量及平均寿命与正常人群比较仍有较大差距[4]–[5]。近些年来，基因治疗在多种遗传性疾病的治疗中都取得了突破。由于血友病B本身属于单基因致病、疗效评价指标明确、基因片段小等特点，使得其成为基因治疗的理想模型。患者有望通过基因治疗获得持续、稳定的自身FⅨ表达，充分改善患者凝血功能，摆脱频繁静脉穿刺，为临床治愈带来希望。

选择合适的FⅨ基因递送载体在血友病B基因治疗中至关重要。经过长期探索，腺相关病毒（AAV）因其非整合性、低免疫原性且递送的基因能够在体内长期表达，而成为当前基因治疗的重要载体[6]–[7]。2011年首次通过静脉注射肝脏靶向AAV载体治疗血友病B的Ⅰ期临床试验取得成功[8]，成为血友病基因治疗探索历程中的一座里程碑。2009年，在一个意大利家族中发现了高活性FⅨ突变体，命名为FⅨ（Padua）[9]。FⅨ（Padua）在相同FⅨ抗原水平时具有更高的FⅨ活性，在基因治疗中可以减少载体用量，成为当前基因治疗FⅨ突变体的主要选择[10]。迄今，血友病B基因治疗领域已成功完成3项Ⅲ期临床试验[11]–[13]，相关产品的基本结构均为AAV-FⅨ（Padua），受试者人群维持了较高的平均FⅨ活性水平，年化出血率和FⅨ制剂的使用都显著减少，无血栓或抑制物发生，部分患者呈现出临床治愈的特征。患者首次有望摆脱疾病负担、回归正常生活。

现有血友病B基因治疗临床试验中，与治疗相关的严重不良事件少见，总体安全性较好。部分血友病B患者接受AAV基因治疗后可能出现无症状转氨酶升高、FⅨ表达降低等肝细胞损伤表现[14]，是影响目的基因长期表达的重要因素之一。自2011年首次观察到阶段性使用糖皮质激素可有效缓解患者肝细胞损伤并可能维持FⅨ表达的作用后，免疫抑制剂在相关试验中得到广泛使用，绝大部分患者在停药后仍能维持FⅨ稳定表达，并且免疫抑制疗法相关的不良反应较轻且短暂，总体效果比较好[11]–[14]。需要强调，目前还没有明确、统一的的免疫抑制治疗方案，因此临床需要根据患者实际情况，结合临床试验所报告的方案，综合制定个体化的免疫抑制治疗方案。另外，现有基因治疗产品的AAV载体发生插入诱变的风险极低，在临床试验中报道的几例发生肿瘤事件经过分析认为与AAV载体无关[15]–[17]。最后，受试者均可在不同时间内清除精液中的AAV基因[11]–[12],[18]，提示AAV基因治疗意外导致患者生殖毒性的风险极低。随着基因治疗逐渐进入临床，真实世界的数据将不断丰富，尚未明确的问题有望逐步解决或得到确证。

在探索血友病B基因治疗的历程中，中国学者取得了骄人的成绩。早在1993年，复旦大学医学遗传研究院就发表了世界首项血友病B基因治疗临床试验，将重组逆转录病毒载体转导的自体成纤维细胞皮下注入患者体内，发现1例患者FⅨ表达至少持续6个月，活性水平由2.9%提高到6.3%，出血症状得到明显缓解[19]，首次概念性观察到基因治疗的前景。随后的一项试验采用类似方法在2例患者体内都改善了FⅨ活性[20]，但其持久性仍不能满足临床需要。最近的突破发生在2022年，中国医学科学院血液病医院报告首个肝脏靶向AAV血友病B基因治疗临床试验取得成功，这也是亚洲首个基因治疗药物的临床研究成果[18]。该试验的成功立足于药物研发和试验设计的双重创新。在药物研发方面，递送载体采用了具有自主知识产权的AAV843血清型衣壳，该载体具有肝脏靶向性强和中和抗体分布低的特征，能够特异性高效感染人肝细胞；表达元件中含有的LXP2.1肝特异性启动子，可以有效促进FⅨ在肝细胞中特异性表达，减少载体的剂量和相关不良反应；与其他同类研究比较，FⅨ表达速度更快，输注后1 d内即观察到FⅨ表达，1周内快速达峰。在试验设计方面，为了更好地抑制AAV引发免疫反应导致FⅨ表达下降或丧失，开创性地采用了在输注前预防性使用糖皮质激素的方案，有效地抑制了载体诱发的免疫反应水平，有利于获得更加持久平稳的FⅨ活性，后续随访超过3年，患者平均FⅨ活性水平仍维持在37%以上。其中1例患者在接受基因治疗后第479天行膝关节置换术，围术期未输注FⅨ药物，成为全球首例在无替代治疗下完成大手术的血友病B患者，验证了该基因治疗能持久改善患者凝血功能[18],[21]，受到国内外同道的高度肯定。截至目前，该治疗方案的注册临床研究也已完成52周随访，结果发布于2024年美国血液学年会ASH[13]。该研究纳入的26例患者接受单次波哌达可基注射后1年平均FⅨ活性维持在55.08 IU/dl，年化出血率降至0.6，且未发现不良事件。该研究进一步证实创造性的“中国基因治疗方案”为血友病B患者提供了新的选择。

随着血友病基因治疗即将进入中国临床，为进一步规范药物的临床使用，由中华医学会血液学分会血栓与止血学组及中国血友病协作组发起，国内二十余位血友病专家共同参与制定了《中国血友病B腺相关病毒载体基因治疗临床应用指导意见（2025）》（下文简称《指导意见》）。《指导意见》是国内在这一领域内的首份指导性文件，在世界范围内同类文件也寥若晨星，成为中国血友病B基因治疗发展过程中的又一座里程碑。《指导意见》为血友病B基因疗法全流程的规范化给出了详细的指导意见，涵盖对机构开展基因治疗的条件配置要求、医患共同做出临床决策的过程、明确基因治疗的适应证和禁忌证、基因治疗产品和伴随药物的选择与使用、基因治疗后的随访事项以及改善患者生活方式的建议等内容。

该文件中的核心内容包括：

①对于国内开展基因治疗医疗机构的要求和建议：未来开展基因治疗的中心需配备生物安全柜、静脉输液泵、监护设备、急救/抢救设备等常规硬件，建议成立由血液科、康复科、药剂科、肝病/消化科、检验科等相关专业医师及血友病护士组成的多学科合作团队，共同参与基因治疗患者的全流程管理。此外《指导意见》中建议医疗机构在首次开展基因治疗之前与有经验的临床研究中心交流学习，提高操作管理的规范性。

②治疗前患者的选择和评估：建议综合评估患者疾病分型和临床表型、年龄、FⅨ抑制物及暴露日、预存AAV抗体检测结果、肝功能情况等，治疗前向患者充分告知风险及获益。

③治疗前及治疗过程中免疫抑制剂使用：患者接受波哌达可基治疗之前，建议所有患者在用药前1天开始应用免疫抑制剂，后规律减量。治疗过程中如出现转氨酶波动或FⅨ水平下降，需结合临床情况增加免疫抑制剂使用。

④治疗后疗效及安全性监测：血友病B患者接受基因治疗后主要监测指标为FⅨ活性和安全性监测。《指导意见》中对于具体监测频率、不同FⅨ活性对应临床意义做出了解释。安全性方面，肝脏健康状态的随访及处理、肿瘤风险的评估、肿瘤风险的随访评估都是需要临床关注的方向。

《指导意见》充分结合了“以患者为中心”和尊重科学的临床理念，符合当下中国血友病B基因治疗临床实践的特点和需要，也将会对其他疾病基因治疗的研究与实践产生深远的影响。相信未来基因治疗的临床落地会给更多患者生活带来质的改变。当然，《指导意见》的局限性在于其内容仅基于国内学者近年有限的循证证据和临床经验而制定。今后，《指导意见》将随着更多研究数据的发布以及基因治疗中国真实世界证据的不断积累而持续更新和完善。
